# Editorial: Frontiers in global regulatory landscape of CRISPR-edited plants

**DOI:** 10.3389/fpls.2024.1367698

**Published:** 2024-01-25

**Authors:** Aftab Ahmad, Neil E. Hoffman, Michael G. K. Jones, Baohong Zhang

**Affiliations:** ^1^Biochemistry/Center for Advanced Studies in Agriculture and Food Security (CASAFS), University of Agriculture, Faisalabad, Pakistan; ^2^Biotechnology Regulatory Services (BRS), United States Department of Agriculture-Animal and Plant Health Inspection Service (USDA-APHIS)-Biotechnology Regulatory Services, Riverdale, MD, United States; ^3^WA State Agricultural Biotechnology Centre, Murdoch University, Perth, WA, Australia; ^4^Department of Biology, East Carolina University, Greenville, NC, United States

**Keywords:** gene editing, CRISPR-Cas, regulation of CRISPR-edited plants, gene edited crops, CRISPR-edited plants

## Emerging applications of CRISPR-Cas systems in plants

CRISPR systems have evolved rapidly for precise gene manipulation in diverse species, including bacteria, plants, animals, and humans. Ashraf et al. describe applications of CRISPR-Cas12a for improving resistance against cotton leaf curl virus diseases. The article demonstrates the successful editing of different genes of the virus using a multiplex CRISPR-Cas12 system to improve resistance against this virus disease. The findings could contribute to developing gene editing strategies for controlling plant virus diseases. Salvagnin et al. provide proof-of-concept gene editing in chicory using different delivery methods of CRISPR reagents. This study highlights the editing efficiency and off-target effects of different delivery methods. Pan et al. demonstrate the establishment of an efficient CRISPR-Cas system for gene editing in lettuce using intron-mediated enhancement (IME)-assisted 35S promoter to express Cas9 and gRNA in a single transcript. IME moderately enhanced expression of both Cas9 and gRNA and thus improved the efficiency of gene editing in lettuce. In addition, the developmental regulator gene GRF5 was also co-expressed with Cas9 and gRNA, which resulted in enhanced editing efficiency. The article demonstrates that both approaches resulted in high editing efficiency in lettuce and can be used to characterize genes in lettuce and other crops. Huffel et al. provide two computational frameworks for an efficient and well-informed design of multiplex gene editing experiments in plants. Both approaches consider different design parameters, such as the number of genes, the number of gRNAs per target site per gene, and resulting editing efficiencies.

## RNPs as CRISPR reagents: an effective strategy to generate transgene free CRISPR crops

Transgene-free CRISPR-edited crops are likely to be more acceptable from a regulatory and public perception standpoint. Kong et al. describe a more efficient positive screening method applicable to diverse plant species, denoted as the PARS (PAR-1 based) strategy for identification of transgene-free CRISPR edited plants using paraquat resistance (PAR-1). The authors demonstrate successful identification of transgene-free edited plants at target loci in the T1 generation. The authors suggest that the PAR-1 strategy could be used to identify transgene-free CRISPR-edited plants in many plant species. Salvagnin et al. compare three different delivery methods - agrobacterium-mediated transformation, transient transfection using plasmids, and RNPs for CRISPR-based gene editing in chicory (*Cichorium intybus* L.). The authors evaluate editing efficiency, off-targets and socioeconomic impact of these methods by editing the germacrene A synthase gene in chicory. The article demonstrates a high editing efficiency of genes with all these delivery methods. Although transient transfection is more convenient due to its simplicity, cost-effectiveness, fewer off-targets and more regenerants, the plasmid-based transient approach led to more plants with permanent integration of plasmid copies. In contrast, RNP transfection of protoplast does not introduce foreign DNA into the plant cell: the authors suggested that RNPs would improve future regulatory and consumer acceptance. Poddar et al. demonstrate applications of RNP-based CRISPR-Cas for editing disease susceptibility genes in wheat. The article demonstrates rapid screening of effective gRNAs using RNPs in wheat protoplast for gene editing in regenerable wheat immature embryos without using a selection marker. In addition, high temperature treatment enhanced gene editing both in protoplasts and immature embryos. The authors highlight the importance of their method for DNA-free gene editing in other crops.

## Regulating CRISPR edited crops

The review of Mendelsohn et al. provides a perspective on regulatory oversight of plant-incorporated pesticides (PIPs) produced using conventional or biotechnological tools. The paper notes that PIPs made using conventional methods are not regulated and that the EPA recently revised their regulations to exempt PIPs developed by genome editing if they otherwise could be generated through conventional methods. Hoffman describes how the USDA revised regulations of gene-edited crops can contribute to achieve sustainable agriculture goals and address challenges associated with climate change. The paper identifies many examples of CRISPR-edited crops that may potentially contribute to these objectives, but most have not been commercialized under the legacy regulations and existing regulatory barriers. In 2020, the USDA revised its regulatory framework for gene-edited crops to make it more science-based, risk-proportionate, and streamlined. The author summarizes how each example might fare under the revised biotechnology regulations, expecting that many more innovations that could contribute to sustainable agriculture goals will be realized under the revised regulations. Ahmad et al. explore the ongoing debate on the regulatory and legal status of CRISPR-edited crops in different countries. The authors highlight the differences in the legal status of CRISPR-edited crops as GMOs or non-GMOs in different countries - the outcomes in the USA and EU will significantly impact future developments of CRISPR-Cas, public perception, and commercialization of edited crops. The authors suggest that the current division as GMOs or non-GMOs of CRISPR crops will persist until a universal, transparent, science-based and scalable regulatory system has been agreed. San-Epifanio et al. review the possible regulatory landscape for the approval of CRISPR edited crops in the EU. The paper highlights that if a proposed regulatory framework for gene-edited crops is approved in 2024, CRISPR-edited crops in the EU will be approved as either crops whose genome has been modified by mutagenesis, cis-genesis, intra-genesis or transgenesis, where regulation would vary depending on the category. However, if the proposed legislation is not approved, CRISPR-edited crops will be considered as GM crops. The authors suggest that although the legislation initiated in 2021 is not sufficient for CRISPR-edited crops, the current framework offers short-term benefits compared to its alternatives. Consequently, the member states should strive for substantial improvements in the legislation for CRISPR-editing and plant breeding in the EU. In an opinion, Forbes et al. explore the possibility of approving genetically modified late blight-resistant potato for commercial cultivation in Norway by emphasizing sustainable considerations during regulation of CRISPR-edited crops in the EU. The authors emphasize that approval would signify a major development in Norway’s stance on GM crops with potential implications on food production and sustainable agriculture. The authors suggest that the EU could adopt a similar approach to address the challenges of sustainable agriculture. This article explores regulatory and public perception challenges of introducing GMOs in Norway. Tachikawa and Matsuo demonstrate that regulatory oversight of gene-edited organisms and products is not yet aligned. The international regulatory landscape is a mosaic, posing challenges for harmonization. The paper demonstrates two regulatory frameworks for gene-edited crops: i) a process-based system which considers gene-edited crops as GMOs, but with simplified regulation, ii) a product-based system, which considers gene-edited crops as non-GMOs but needs confirmation. The article explores the reason behind the tendency of convergence between these two approaches to regulate CRISPR-edited crops, and examines the challenges and implications of these approaches in governance of the agriculture and food sector in the context of gene-editing.

## Author contributions

AA: Writing – original draft. NH: Writing – original draft, Writing – review & editing. MJ: Writing – original draft, Writing – review & editing. BZ: Writing – original draft, Writing – review & editing.

